# A straightforward protocol to sample morphological traits of dragonflies and damselflies in the field

**DOI:** 10.1002/ece3.11604

**Published:** 2024-06-23

**Authors:** Roberto Novella Fernandez

**Affiliations:** ^1^ Department for Life Science Systems, School of Life Sciences Technical University of Munich, Terrestrial Ecology Research Group Freising Germany; ^2^ Department of Global Change Ecology, Biocenter University of Würzburg Würzburg Germany

**Keywords:** Anisoptera, body mass, body size, damselflies, dragonflies, field sampling, morphology, Odonata, traits, wing area, wing loading, wing morphometry, Zygoptera

## Abstract

Scarcity of morphological data limits the potential of functional ecology approaches, which rely on traits to elucidate ecological processes. Dragonflies and damselflies (Odonata) are a frequently used ecological model for which, however, only limited morphological data is available. Here, it is presented a field sampling protocol to collect ecologically relevant yet largely unavailable morphological traits of Odonata. The protocol enables the straightforward collection of traits from living individuals directly in the field. Those traits include body mass, wing area and wing loading as well as thorax width, hindwing length and body length. Furthermore, the protocol allows for posterior wing morphometric analyses. The protocol proved to be robust and universally applicable based on testing on roughly half (76) of all European odonate species. The use of this protocol can increase our understanding of odonatan morphology at interspecific and intraspecific levels and assist in developing mechanistic understanding of their ecology.

## 1 INTRODUCTION

Morphological data have recently been increasingly used in ecology due to the rising popularity of functional approaches, which use traits to understand community assembly and biogeographic patterns (Díaz & Cabido, 2001; Violle et al., 2014). Availability of morphological data across species is, however, highly limited, particularly for non‐vertebrate groups such as insects (Wong et al., 2019), in which often only a few measurements from the fauna of well‐studied regions are available. Representativity of such data is, moreover, often uncertain given that species' morphologies vary within and across populations in space and time. The dedicated collection of biologically relevant and traceable morphological data is, thus, pointed out as a key aspect to improving our capacity to understand ecological processes and predict future changes (Wong et al., 2019). For this, standardised data collecting methods are a pre‐requisite.

The Odonata (dragonflies and damselflies) are one of the best‐studied insect taxa, with a rich natural history and extensive observation record track. Moreover, Odonata are frequently used as ecological models due to their responsiveness to habitat and climate changes (Córdoba‐Aguilar, 2008). Although the morphology of Odonata has been extensively studied (Tillyard, 1917), the availability of quantitative and comparable morphological data that can be used in ecological research is highly restricted. Even in the best‐studied regions such as Europe and North America, publicly available traits across species are generally limited to body length, wing length and abdomen length (Aromaa et al., 2019; Harabiš & Hronková, 2020; Waller et al., 2019). These are typically single ‐averaged‐ or ranged values per species, sourced from unreferenced literature, and therefore represent an untraceable sample of museum specimens of uncertain size and origin (Harabiš & Hronková, 2020). Recent ecological studies use this data (Novella‐Fernandez, Brandl, et al., 2023; Novella‐Fernandez, Chalmandrier, et al., 2023; Pinkert et al., 2017; Zeuss et al., 2014), while very few exceptions sample new morphological data from current populations (Grabow & Rüppell, 1995; Worthen & Jones, 2006). Collection of traceable morphological data of odonates is essential to ensure representativity, opens the door to a new range of research possibilities across study systems that require considering morphological variation across environmental gradients and time, and enables ecological inferences based on morphological traits requiring living individuals.

Among animals' morphological traits, body size is probably the single most ecologically relevant one because it relates to multiple dimensions of species' performance and ecological niches (Peters, 1986; Schmidt‐Nielsen, 1984). In Odonata, it links to thermoregulation (May, 1976), energetic requirements or habitat use (Worthen & Jones, 2007), among others. Body mass is the most informative measure of body size, but it is only available for a handful of odonate species (Aromaa et al., 2019). Thus, most ecological studies use more indirect proxies, such as wing length or body length (Oliveira‐Junior et al., 2021; Wonglersak et al., 2020), derivate body volume from scientific illustrations (Pinkert et al., 2017) or estimate body mass using allometric regressions from known species (Aromaa et al., 2019). The limited availability of odonate body mass data originates, in part, from logistic restrictions. An accurate measurement requires living individuals due to changes in water content after death, but transporting living odonates to laboratory facilities poses certain challenges and may lead to biases due to altered animal condition (Grabow & Rüppell, 1995). Likewise, measuring body mass of such small animals directly in—unstable—field conditions is not straightforward. The few previous studies used laboratory scales (Grabow & Rüppell, 1995; Worthen & Jones, 2006), necessarily limiting its applicability and do not detail how to ensure reliable readings in the field.

Besides body size, Odonata species' ecology is strongly influenced by traits determining flight performance because all odonatan activity requires flight. For instance, wing size affects manoeuvrability and size of anal triangle improves gliding (Bomphrey et al., 2016), altogether having implications on species' ecology (Johansson et al., 2009; Sacchi & Hardersen, 2013; Suárez‐Tovar & Sarmiento, 2016). The relation between body mass and wing area, defined as wing loading, is another essential trait defining performance in flying animals. Species with a high wing loading exhibit fast and thus less maneuverables flight than those with a low wing loading (Wainwright & Reilly, 1994). In better‐studied aerial animals such as birds and bats, species' wing loading relates to habitat use and trophic ecology (Norberg & Rayner, 1987). Wing loading requires both wing area and body mass measurements from living individuals. Without current practical guidance on the collection of either, this trait is only available for a few species (Grabow & Rüppell, 1995; Worthen & Jones, 2006), and its role driving species´ performance and ecology is, thus, so far poorly explored.

The primary function of Odonata's thorax is hosting the flight musculature (Bäumler et al., 2018), and therefore its size is expected to link to species´ flight performances, influencing their biology and ecology. Data on odonate's thorax sizes across species is, however, scarce (Powney et al., 2014), and consequently, it is poorly understood how it may drive species' performance, biology and diversity patterns.

Here, it is presented a morphological sampling protocol that aims to describe the standard collection, directly in the field and from living individuals, of several key morphological traits determining Odonata biology: body mass, thorax width, wing loading, wing area, as well as hindwing length and body length. First, the material required and the protocol itself are described, and then the suitability of each measurement for the biological and ecological understanding of Odonata is discussed based on experience of testing the protocol on approximately half of all European species.

## 2 MATERIAL

**FIGURE 1:**
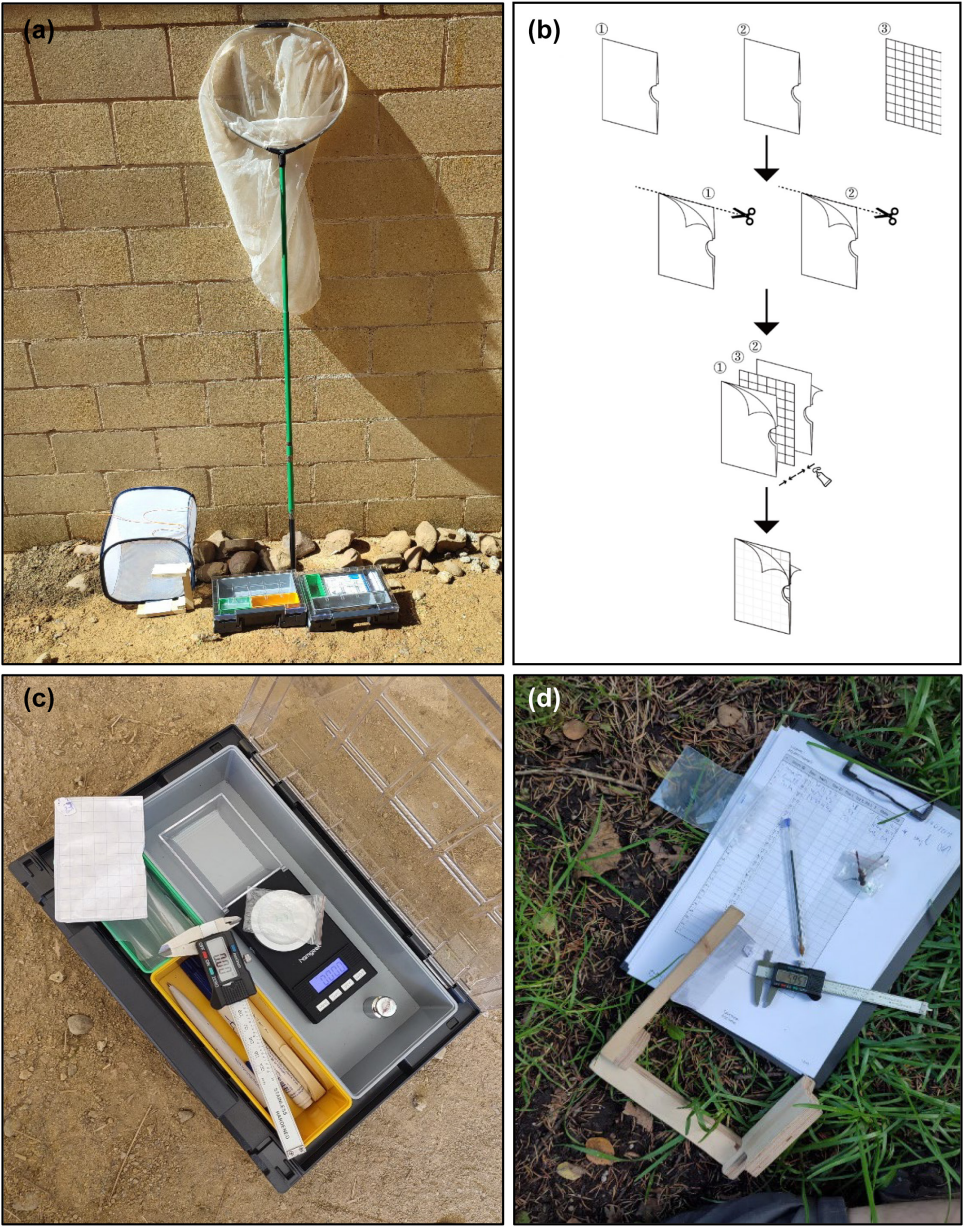
(a) The material required to sample morphological traits of odonates fits in a rucksack. From left to right: folding cage, cardholder frame, toolboxes, aerial net. (b) Process to build the modified the cardholder for wing photographs. (c) Digital scale and weighting box, calliper, modified cardholder and plastic bag. (d) Cardholder frame, calliper, plastic bag with odonate individual to be weighted.

*Entomological aerial net*: 40 cm rim diameter with 1.5 m handle is suited for most species, while for the largest members of Aeshnidae and Cordulegastridae, 50 cm rim diameter and >2 m handle is most adequate. Carbon fibre nets are lighter weight and therefore preferred (Figure 1a).

*Foldable mesh cage*: Can be bought as *butterfly cage* or *insect‐rearing cage*. They are lightweight, and several of them can easily fit in a backpack. A strap can be added for better handling and to enable attaching them to vegetation (Figure 1a).

**FIGURE 2:**
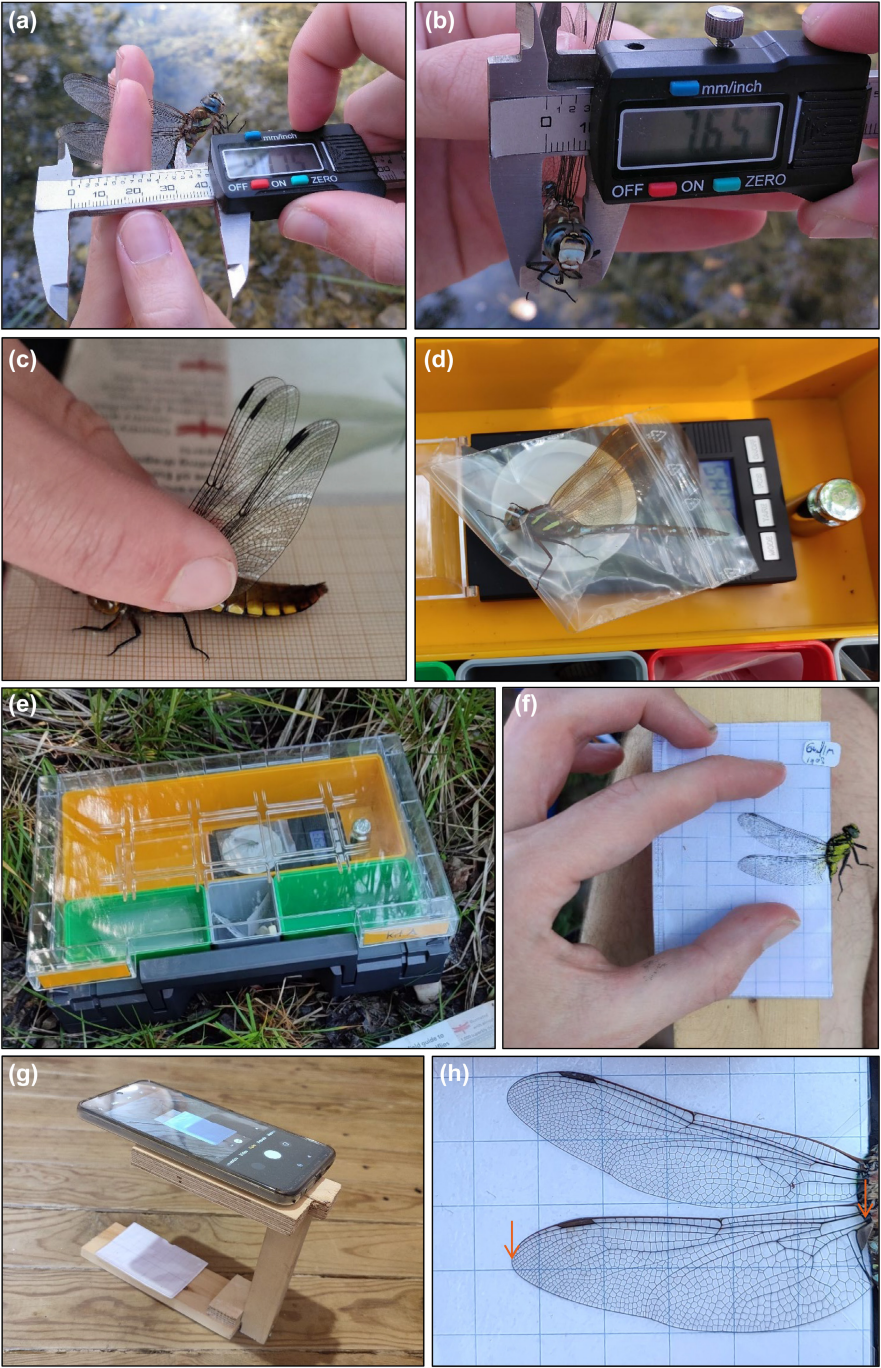
(a) Hindwing length is measured from the apex to the basal insertion of the median vein (arrows in h) using a calliper. (b) Thorax width is measured using a calliper in diagonal between the head and the wings. Note correct handling in a and b. (c) Body length is measured using a sheet of gridded paper. (d) Body mass is measured by placing the individual in a plastic bag and using the scale within a weighting box. (e) Lid must be closed for each reading to prevent wind disturbances. (f) Wing photographs are obtained by introducing individuals´ wings in the respective folds of the modified cardholder. (g) The cardholder with the individual is placed in the cardholder frame to take a standard photograph (f) using a smartphone. (h) Resulting standardised wing image for posterior processing.

*Digital scale*: A small, lightweight and inexpensive battery‐powered portable scale with an accuracy of 0.002 g can easily be found online from different brands, usually marketed as a *precision jewellery scale* (Figures 1c and 2d,e).

*Weighting box*: Box with transparent lid that allows scale readings without wind disturbances. An assortment box 44 × 35.5 cm of the brand Auer (Figure 1c,e) was particularly suitable because compartment distribution can be configured.

*Small plastic bags to weight individuals*: Different sizes are suited for different species (Figures 1c,d and 2d). They can be clipped to facilitate placing the individuals inside.

*Modified cardholder for wing photographs*: Built from two transparent cardholder cases and a sheet of 8 mm gridded paper. See a diagram with building instructions in Figure 1b. First, the top margin of cardholders is cut so that they can be opened from both, right and top sides. Then, the two cardholders are pasted (*Instant Glu‐xtreme Brico*) to each side of the gridded paper sheet. The resulting structure (Figures 1c and 2f,g) has two transparent folds opening from the lateral and top sides which will accommodate each respective pair of wings (left and right) of the odonate. The gridded paper will later serve as a background reference area of known size and will allow addressing possible image geometric deformation with software. A small semi‐circular incision can be made in the right margin of the central gridded paper sheet to help accommodating the thorax of the individual. Alternative cardholders with slightly different incisions can be built for differently sized species.

*Cardholder frame for standardised wing photographs and smartphone*: A simple self‐made wooden structure consisting of two plates that will hold, respectively, the cardholder and a smartphone connected by with an arm (Figures 1d and 2f). The arm has certain length and is attached to the plates at certain angle that allows perpendicular photographs minimising geometric distortion. The specific configuration will depend on the smartphone model.

*Gridded surface*: 1 mm gridded paper sheet can be pasted on a flat surface such as a notebook (Figure 2c).

*Common office material*: a calliper, 1 mm gridded paper, pens and markers (Figure 1d).

## 3 PROCEDURE

Individuals are captured using the entomological aerial net, with a quick movement directed from behind or below the animal. Hawker species such as the Aeshnidae members, which fly continuously, may pose certain challenges. For these, sampling in small—more accessible—aquatic habitats over which individuals sometimes fly in repetitive patterns gives best results. Once captured, individuals must be handled carefully by grasping and closing their four wings (Figure 2a–c) and placed inside the mesh foldable cages until they can be processed.

Individuals of different species can be placed in the same mesh cage unless they differ greatly in size, but high densities should be avoided because it can result in the damage of individuals. In hot and sunny conditions, mesh cages should be kept away from direct sun to prevent individuals from overheat. Long pre‐processing periods in mesh cages may be avoided if aiming to measure body mass accurately, for example, six individuals of *Somatoclora flavomaculata* lost weight at an average rate of 1.67 ± 0.1% per hour, likely from dehydration while deprived from foraging.

Processing of individuals starts with identifying its species, sex and rough developmental class, the latter being relevant because body mass changes along development. For this, a categorisation among teneral (recently emerged), immature, mature and old is generally possible. Tenerals are easily told apart by their unrigid cuticula and wings and weak flight. Immature and mature individuals can, very often, be distinguished based on their species‐specific colouration patterns, which are described in specialised guides (Dijkstra et al., 2006). Finally, old individuals can typically be distinguished by their darker colouration and sometimes tattered appearance, including damaged wings and missing tarsae.

Once identified and sexed, morphological measurements of individuals follow:

*Hindwing length*: For a right‐handed researcher, the left hand is used to hold all four wings between index and middle fingers, placing the odonate in a natural dorso‐ventral position facing either left or right (Figure 2a) depending on the wing to be measured. Hindwing length can be measured using a calliper from the ventral side, from the apex of the wing to the insertion of the median vein at the wing base (Figure 2h) for species whose size allows usto find this structure. For small damselflies, hindwing length can be more simply measured by using the cardholder (see “Standardised wing photographs”).

*Thorax width*: For a right‐handed researcher, left hand is used to hold all four wings with thumb and index fingers placing the odonate facing towards oneself. A calliper is carefully introduced in angle between the animals´ head and front wings, and its thorax is measured by its thickest area (Figure 2b).

*Body length*: Individual held by its four wings is placed on top of 1 mm gridded surface with its abdomen fully stretched. The measure from its head (frons) to the end of its abdomen (segment 10) is taken without considering anal appendixes (Figure 2c).

*Body mass*: The weighting box with the scale is placed on flat and stable ground under shade. Note that ground stability is essential for accurate readings and can be critical in unstable habitats such as peatlands. Stability sometimes can be improved by clearing vegetation remains. The use of a portable camping table is not recommended because it will compromise stability. Once the weighting box is placed in suitable ground, the scale must be calibrated using a calibrated weight and not moved until the end of the measurements. Then, the individual is carefully placed with its wings folded inside the plastic bag of suitable size and weighted (Figure 2d), closing the lid of the weighting box for each reading to prevent wind disturbances (Figure 2e). Given the unstable nature of field conditions, readings must be validated by ensuring that exact measures of known calibrated weights are given before and after each sample. Note that individuals should not be kept within the plastic bag for longer than the few seconds needed.

*Standardised wing photographs*: Individuals are carefully held by the ventral side of their thorax with thumb and index fingers, partially covering the base of its wings, which allows to close them partially. In this position, both wing pairs (left and right) are carefully introduced in each respective fold of the modified cardholder, which immobilises the individual (Figure 2f). Wings can, then, be carefully set within the cardholder so that they are completely visible and do not overlap (Figure 2f). Then, the cardholder and smartphone are placed in the cardholder frame (Figure 2g) to obtain standardised photographs of a pair (left or right) of wings in terms of distance, angle and limit geometric distortions (Figure 2f,h). Individual's id may have been previously added with a sticky label to the cardholder (Figure 2f). Note that for the smallest damselflies, hindwing length can also be measured while in the cardholder using the calliper.

After the measurements, individuals can be released unharmed in the same location. They can optionally be marked on the wing using a permanent marker to prevent repeated captures.

Once out of the field, standardised wing photographs can be processed digitally. Wing area of fore and hind wings can be obtained with imaging software (e.g. ImageJ) based on the relation between the number of pixels within the area of the wing and certain gridded reference area from the background:

$$\text{Area of}\;a\;\text{wing}\;\left({\mathrm{cm}}^{2}\right)=\frac{\text{Area reference}\;\left({\mathrm{cm}}^{2}\right)}{\text{Pixels reference}\;}\times \text{pixels wing}.$$

The wing area of the individual is the sum of all four wing areas:

$$\text{Wing area}\;\left({\mathrm{cm}}^{2}\right)=2\;\left(\text{area forewing}+\text{area hindwing}\right).$$

The wing loading of the individual corresponds to its body mass divided by its wing area:

$$\text{Wing loading}\;\left(\mathrm{mg}/{\mathrm{cm}}^{2}\right)=\frac{\text{Body mass}\;\left(\mathrm{mg}\right)}{\text{Wing area}\;\left({\mathrm{cm}}^{2}\right)}.$$

Additionally, wing photographs allow for morphometric analyses.

## 4 RESULTS AND DISCUSSION

While scarcity of recent traceable morphological data limits inferences from functional ecology approaches, there is currently no practical guidance on the collection of morphological traits of Odonata. This sampling protocol details the methods to measure, accurately and in a standard manner, several ecologically relevant morphological traits from living odonates, directly in the field and using simple logistics. The protocol proved robust, as it was tested on 76 European dragonfly and damselfly species of all families and sizes: from the smallest *Nehalenia speciosa* to the largest *Anax imperator*, and enabled measuring biologically essential yet rarely available species´ traits such as body mass, wing area and wing loading, as well as allowing posterior wing morphometric analyses. Processing time ranged between 3 and 7 min per individual. The main benefit of this protocol with respect to methods used in previous studies is its simple logistics. Traits can be measured by a single person using few and inexpensive equipment without generating posterior laboratory work and avoiding the ethical, conservation and bureaucratic problems from methods requiring killing individuals. Therefore, this protocol is suitable to be widely applied, even by non‐professional odonate researchers. Based on experience in the field, the use of this protocol can increase our understanding of odonatan morphology across faunas at interspecific and intraspecific levels, which can facilitate understanding species´ biology, ecomorphology and ecological niches, and the diversity patterns of the group. Discussion on specific measurements follows.

The most commonly available morphologic measurements in odonates are hindwing length and body length. The measure of hindwing length proposed proved accurate, quick and straightforward. Conversely, the method proposed to measure body length can be useful to describe general species' morphology, but it would hardly provide accurate measurements to assessing fine intraspecific differences because of the great mobility of this body region and its regular expansion and contraction due to abdominal ventilation. The equivalent measure from preserved museum specimens is, however, unlikely to be more accurate because rigidly preserved specimens can hold a variety of positions, which difficults accurate measurements.

Thorax size is a scarcely available morphological trait for the Odonata. The proposed measurement of thorax width proved accurate, quick and reliable. Its collection, therefore, is encouraged as it could reveal novel aspects of odonatan biology and ecology. In sampling designs where the more complex measure of body mass is not feasible, either thorax size or wing length could be used as a proxy for individuals' body size.

Body mass of Odonata species is generally unknown due to practical complexities in its measure. Following this protocol can enable obtaining accurate and repeatable readings of body mass of living adult dragonflies and damselflies in field conditions. Note, however, that reliable readings require careful manipulation, placing the weighting box on stable ground, calibration, closing the lid and validating before and after each reading with a calibrated weight. Measurement error in these conditions was, based on calibrated weights, estimated at around ~4 mg. This method, therefore, can be used to describe fine intraspecific differences across and within populations, particularly for the larger dragonflies (~80–1000 mg) in which measuring error is relatively smaller. Moreover, for large species, it may also enable assessing intraindividual body mass changes in capture‐recapture studies, allowing to inform multiple understudied aspects of Odonata biology, such as changes along individual's development due to changes such as flight muscle, fat reserve, reproductive or overall status (Corbet, 2004). For the smaller damselflies, values recovered using this protocol will have much lower relative accuracy, particularly the smallest species in which body mass can be as little as ~20 mg. For those, this method can be of more limited use and be relegated to maybe rough interspecific comparisons.

The proposed method to obtain standardised wing images proved very effective provided careful manipulation. The images obtained allowed for posterior measures of wing area and wing loading of individuals, which allow characterising species accurately and also assess fine intraspecific differences. Furthermore, wing images can enable assessing wing morphology, both across species (Chitsaz et al., 2020; Johansson et al., 2009) and within species, which is rarely addressed (Hassall, 2015; Pinto et al., 2012).

In conclusion, the combined accurate obtention of biologically relevant morphological traits and uncomplicated logistics of this protocol can assist in developing further our morphological understanding of Odonata. While specifically designed for odonates, this protocol may likely be in part adapted to other insect taxa, for instance, the scale within the weighting box for body mass or the cardholder for wing photographs. I hope that this protocol will stimulate the collection of morphological data for odonates and other insects and promote functional ecological research.

## AUTHOR CONTRIBUTIONS

**Roberto Novella Fernandez:** Conceptualization (lead); investigation (lead); methodology (lead); writing – original draft (lead).

## FUNDING INFORMATION

No funding was received to carry out the research described in this manuscript.

## CONFLICT OF INTEREST STATEMENT

The author declares no conflict of interests.

## Data Availability

No data were used in this manuscript.
